# Effects of 1-Methylnicotinamide (MNA) on Exercise Capacity and Endothelial Response in Diabetic Mice

**DOI:** 10.1371/journal.pone.0130908

**Published:** 2015-06-26

**Authors:** Kamil Przyborowski, Marta Wojewoda, Barbara Sitek, Agnieszka Zakrzewska, Agnieszka Kij, Krystyna Wandzel, Jerzy Andrzej Zoladz, Stefan Chlopicki

**Affiliations:** 1 Jagiellonian Centre for Experimental Therapeutics (JCET), Jagiellonian University, Krakow, Poland; 2 Department of Pharmacokinetics and Physical Pharmacy, Jagiellonian University Medical College, Krakow, Poland; 3 Department of Muscle Physiology, Faculty of Rehabilitation, University School of Physical Education, Krakow, Poland; 4 Department of Experimental Pharmacology, Jagiellonian University Medical College, Krakow, Poland; UFMG, BRAZIL

## Abstract

1-Methylnicotinamide (MNA), which was initially considered to be a biologically inactive endogenous metabolite of nicotinamide, has emerged as an anti-thrombotic and anti-inflammatory agent with the capacity to release prostacyclin (PGI_2_). In the present study, we characterized the effects of MNA on exercise capacity and the endothelial response to exercise in diabetic mice. Eight-week-old db/db mice were untreated or treated with MNA for 4 weeks (100 mg·kg^-1^), and their exercise capacity as well as NO- and PGI_2_-dependent response to endurance running were subsequently assessed. MNA treatment of db/db mice resulted in four-fold and three-fold elevation of urine concentrations of MNA and its metabolites (Met-2PY + Met-4PY), respectively (P<0.01), but did not affect HbA_1c_ concentration, fasting glucose concentration or lipid profile. However, insulin sensitivity was improved (P<0.01). In MNA-treated db/db mice, the time to fatigue for endurance exercise was significantly prolonged (P<0.05). Post-exercise Δ6-keto-PGF_1α_ (difference between mean concentration in the sedentary and exercised groups) tended to increase, and post-exercise leukocytosis was substantially reduced in MNA-treated animals. In turn, the post-exercise fall in plasma concentration of nitrate was not affected by MNA. In conclusion, we demonstrated for the first time that MNA improves endurance exercise capacity in mice with diabetes, and may also decrease the cardiovascular risk of exercise.

## Introduction

1-methylnicotinamide (MNA) is major product of nicotinamide (vit B_3_, PP) metabolism, and is generated by nicotinamide N-methyltransferase (NNMT) and then further converted into 1-methyl-2-pyridone-5-carboxamide (Met-2-PY) and 1-methyl-4-pyridone-5-carboxamide (Met-4-PY) [1, 2]. It has been reported that MNA is a biologically active compound, and experimental studies in *in vivo* animal models have demonstrated that the anti-thrombotic [3], anti-inflammatory [4] and gastroprotective [5] effects of MNA are mediated by a prostacyclin (PGI_2_)-dependent mechanism. Additionally, chronic administration of MNA in animal models of hypertriglyceridemia and diabetes resulted in improvement of nitric oxide (NO)-dependent endothelial function [6].

It is well known that PGI_2_ production is increased during exercise [7, 8] and PGI_2_ release from the vascular endothelium in response to exercise appears to be an important factor regulating exercise tolerance and exercise capacity [9]. Furthermore, Zoladz et al. [9] have suggested that impairment of the exercise-induced release of PGI_2_ may be responsible for the increased cardiovascular risk of vigorous exercise. Since it has been reported that diabetic patients have decreased ability to release PGI_2_ during exercise [10], and are characterized by higher cardiovascular risk during vigorous exercise [11] pharmacological stimulation of post-exercise PGI_2_ production may prove beneficial.

NO is also involved in the regulation of exercise capacity, and NO generated by NO synthase is metabolized in the body to inorganic anions: nitrite (NO_2_^-^) and nitrate (NO_3_^-^) [12]. On the other hand, nitrite may be reduced back to NO by enzymatic and non-enzymatic pathways, particularly in acidic environments with low oxygen availability [12], which occurs during exercise [13]. It has been reported that single bout of strenuous physical exercise had no effect on plasma nitrate concentrations in humans [14]. However, others have demonstrated a small post-exercise increase in plasma nitrate concentrations [15] or increase in plasma nitrite concentrations [16]. Furthermore, exogenous nitrate and the subsequent increase in plasma nitrite concentrations was accompanied by enhanced exercise tolerance in humans [17]. Thus, enhanced NO bioavailability appears to enhance exercise capacity in humans.

We previously showed that endogenous MNA was involved in the regulation of exercise capacity, since the NNMT-MNA pathway was activated by a single bout of strenuous exercise, with an elevated post-exercise plasma concentration of MNA [18]. Considering the pharmacological profile of MNA, including PGI_2_ release and improvement of NO-dependent function, one could speculate that MNA supplementation could improve exercise capacity in diabetics and therefore, could be considered as a protective agent against cardiovascular risk during physical activity.

Accordingly, the aim of this work was to characterize the effects of MNA supplementation on exercise capacity and endothelial-, PGI_2_- and NO-dependent response to exercise in diabetic db/db mice. For this purpose, db/db mice were treated with MNA in drinking water (100 mg. kg^-1^) for 4 weeks, their exercise capacity during an endurance running test and post-exercise MNA, nitrite, nitrate and 6-keto-PGF_1α_ concentrations were subsequently assessed.

## Materials and Methods

### Animals

Male C57BL6/J^db/db^ mice (henceforth referred to as db/db mice) purchased from Charles River Laboratories were housed with five mice per cage and a 12-hours light/dark cycle. Animals had free access to drinking water and standard rodent chow. All procedures involving animals were approved by the Local Bioethics Committee in Krakow, Poland (Permit Number: 914/2012; 127/2014) and conducted in accordance with the institutional guidelines.

### Experimental protocol

**Fig 1:**
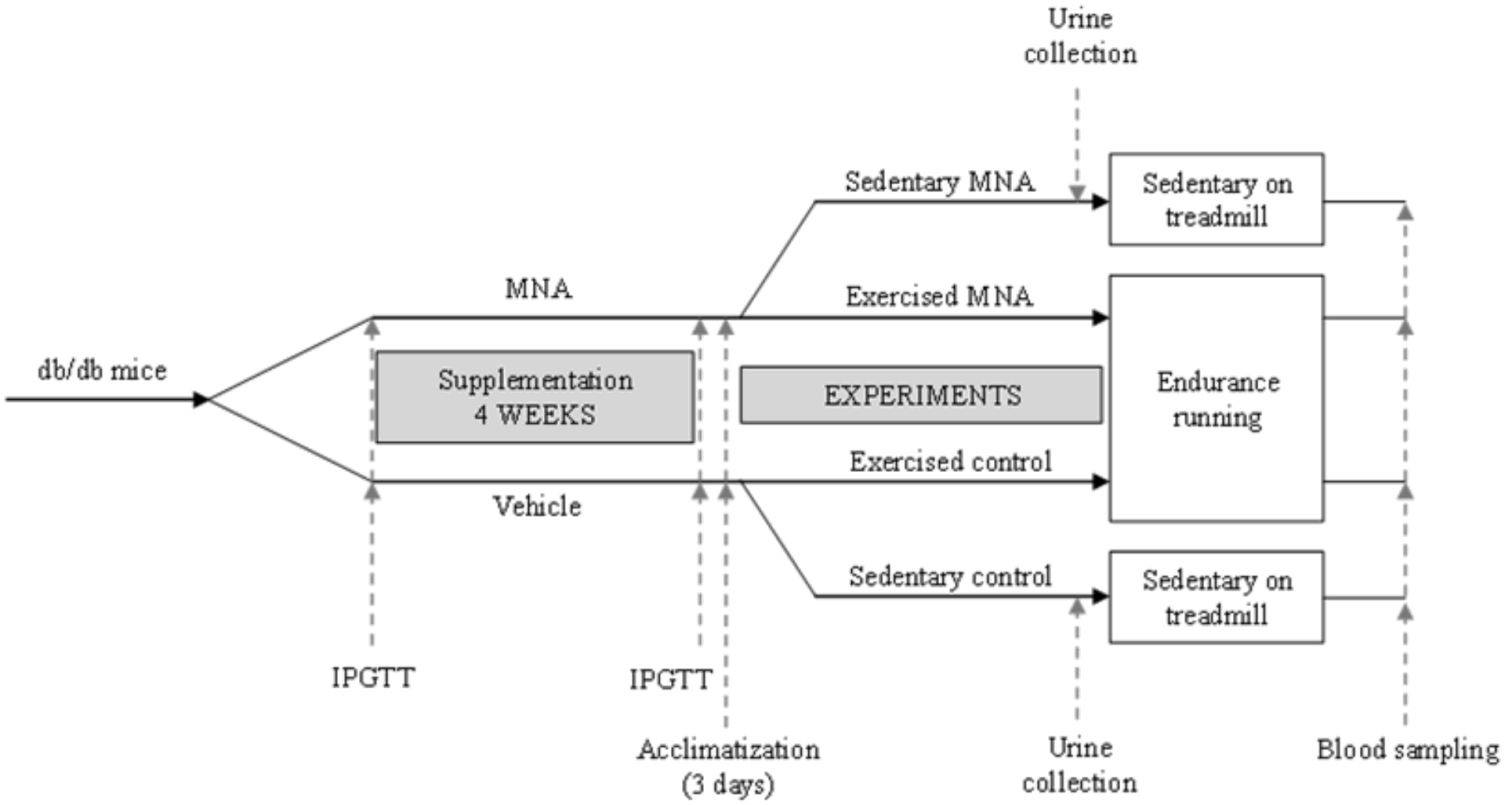
Diagram depicting experimental protocol. Briefly, 8-week-old db/db mice were randomly assigned into four experimental groups: sedentary, exercised mice treated with MNA (sedentary or exercised MNA) and sedentary, exercised mice not treated with MNA (sedentary or exercised control). See text for details of the experimental protocol.

The scheme of the protocol is presented in Fig 1. 8-week-old db/db mice were randomly assigned into the following experimental groups: sedentary or exercised mice not treated with MNA (sedentary or exercised control) and sedentary or exercised mice treated with MNA (sedentary or exercised MNA). MNA was given in drinking water for 4 weeks at a dose of 100 mg·kg^-1^. Mice were weighed once a week in order to adjust the MNA dosage. After 4 weeks of MNA supplementation, the animals assigned into the exercised groups were subjected to endurance running tests as described below.

For the assessment of their running performance capacity, a closed two-line treadmill equipped with an electrode was used (Columbus Instruments, Columbus, OH, USA). Three days before the exercise experiment, the mice were acclimatized. On the first and second day of acclimatization, the mice were placed on the immobile treadmill for 5 min; on the third day, they spent 5 min on the immobile treadmill, followed by 10 min of walking at a velocity of 5 m·min^-1^. The exercise capacity of the db/db mice supplemented and non-supplemented with MNA was evaluated by measuring their endurance running time on the treadmill at 5° incline. The treadmill was started at 5 m · min^-1^ and the speed was incrementally increased by 1 m · min^-1^ every 2.5 minutes to a final velocity of 8 m · min^-1^. The animals were run on the treadmill until they reached fatigue, which was defined as when they being unable to keep running for at least 10 s despite electrical stimulation (current 0.34 mA, voltage 25 V, electrical stimulation frequency 3 Hz). The time from start to finish was recorded. Simultaneously, sedentary mice were placed on the immobile treadmill. The endurance exercise protocol described above was established in the preliminary study.

Immediately after completion of endurance running to fatigue, the mice were anaesthetised with pentobarbital (50 mg·kg^-1^, i.p.), and blood samples were then taken from the right heart ventricle on EDTA-anticoagulant and centrifuged (1000 x g, 10 min, 4°C) to obtain plasma samples. After blood sampling, the mice were euthanized with an excessive dose of pentobarbital (100 mg·kg^-1^, i.p.). Plasma samples were deep frozen (-80°C) and stored until further analysis.

Furthermore, one day before the endurance running test, mice from the sedentary control and MNA groups were placed in individual metabolic cages for 24 h urine sample collection. Urine samples were deep frozen (-80°C) and stored until further analysis.

### Intraperitoneal Glucose Tolerance Test (IPGTT)

4-h-starved male db/db (C57BL6/J^db/db^) mice were given an intraperitoneal injection of glucose (2 g·kg^-1^). Tail blood samples were collected on anticoagulant (trisodium citrate, 3,8%, 9:1) at 0, 15, 30, 45, 60 and 120 minutes after glucose load and subsequently centrifuged (10 000 rpm for 5 minutes at 4°C) to obtain plasma samples. Plasma glucose levels were determined using a biochemical analyser (ABX Pentra 400, Horiba, Germany). IPGTT was performed for all mice before (at 8 weeks of age) and after (at 12 weeks of age) supplementation with MNA.

### Blood cell count and biochemical parameters

Blood cell count was assessed by an Animal Blood Counter (ABC Vet, Horiba, Germany). Biochemical parameters including glucose, HbA_1c_, creatinine concentrations and lipid profile were determined using automatic biochemistry analyser (ABX Pentra 400, Horiba, Germany).

### Measurement of MNA, Met-2PY and Met-4PY

Concentrations of MNA, Met-2PY and Met-4PY in plasma and urine samples were determined using the LC/MS/MS method. Prior to analysis, plasma samples were deproteinized with acidified acetonitrile. Chromatographic analysis was performed on an UltiMate 3000 HPLC system (Thermo Scientific Dionex, Sunnyvale, CA, USA). Chromatographic separation was carried out on an Aquasil C18 analytical column (4.6 mm x 150 mm, 5 μm, Thermo Scientific, Waltham, MA, USA). The mobile phase consisted of acetonitrile (A) and water (B), with the addition of 0.1% formic acid. The flow rate was set at 0.8 ml·min^-1^ with isocratic elution (A:B, 20/80).

Urine samples were diluted 1:10. HPLC analysis was performed on the Transcend TLX-2 system with an HTS PAL System autosampler (Thermo Scientific). Compounds were separated from the matrix using a TurboFlow Cyclone-P polymer column (0.5 x 50 mm, Thermo Scientific). From the TurboFlow column, the analytes were eluted with acidified acetonitrile onto an Aquasil C18 4.6 x 150 mm, 5μm analytical column. The mobile phase consisted of acetonitrile (A) and acidified water (0.1% formic acid) (B) with the following linear eluting steps: 0.0 min (A:B, 80/20)– 1.5 min (A:B, 80/20)– 5.5 min (A:B, 50/50) –6.5 min (A:B, 50/50)– 7.0 min (A:B, 80/20)– 10.0 min (A:B, 80/20). The flow rate was set at 0.8 ml·min^-1^.

Detection was performed on a TSQ Quantum Ultra triple quadrupole mass spectrometer (Thermo Scientific) equipped with a heated electrospray ionization interface (HESI II Probe) operating in the positive ion mode. Data acquisition and processing were accomplished using Xcalibur 2.1 software.

### Measurement of 6-keto-PGF_1α_, nitrite and nitrate in plasma

For the measurement of PGI_2_, the plasma concentration of its stable metabolite 6-keto-PGF_1α_ was determined using a commercially available ELISA kit according to the manufacturer’s instructions.

The concentration of nitrite and nitrate were measured using ENO-20 –NOx Analyzer (Eicom Corp., Kyoto, Japan). The ENO-20 uses a liquid chromatography method with post-column derivatisation using Griess reagent. Nitrite and nitrate were separated from other substances in matrices on an NO-PAK column, 4.6x50mm (Eicom Corp.). Nitrate was reduced to nitrite using a cadmium-copper column (NO-RED, Eicom Corp.). Nitrite was mixed with Griess reagent to form a purple azo dye in a reaction coil placed in a column oven at 35°C, and the absorbance of the dye product was measured at 540 nm. The flow of the mobile phase (Carrier Solution) was 0.33 ml·min^-1^. The Griess reagent (Reactor A and B Solution) was delivered by the pump at a rate of 0.11 ml·min^-1^. The plasma sample was precipitated with methanol at a ratio of 1:1 (v/v), and centrifuged at 10 000 x g for 10 min, and the supernatant was used for analysis.

### Chemicals and drugs

Pentobarbital (Vetbutal, Biowet, Pulawy, Poland), trisodium citrate dihydrate and ethylenediaminetetraacetic acid (EDTA) were obtained from Sigma-Aldrich (St. Louis, MO, USA). N-methyl-4-pyridone-3-carboxamide (Met-2-Py), N-methyl-2-pyridone-5-carboxamide (Met-4-Py) and deuterated internal standard (Met-2-Py-d3 and Met-4-Py-d3) were purchased from TLC PharmaChem (Vaughan, Ontario, Canada). 1- methylnicotinamide chloride and MNA-d3 were kindly provided by Dr. Adamus from the Technical University in Lodz, Poland. HPLC gradient grade acetonitrile, HPLC gradient grade methanol and formic acid were purchased from Sigma-Aldrich. Ultrapure water was delivered by the MiliQ Water Purification System from Merck (Darmstadt, Germany). Ready-to-use reagents for blood cell count and for biochemical parameters determination were used in the study (Horiba, Germany). 6-keto-PGF_1α_ ELISA kit was purchased from ENZO Life Science (Farmingdale, NY, USA).

### Statistical analysis

Statistical analysis was performed using GraphPad Prism 5. The area under the curve (AUC) was calculated using the trapezoidal rule in Microsoft Excel. P values < 0.05 were considered as statistically significant.

## Results

### Effects of MNA treatment on glucose and lipid profile in db/db mice

**Fig 2:**
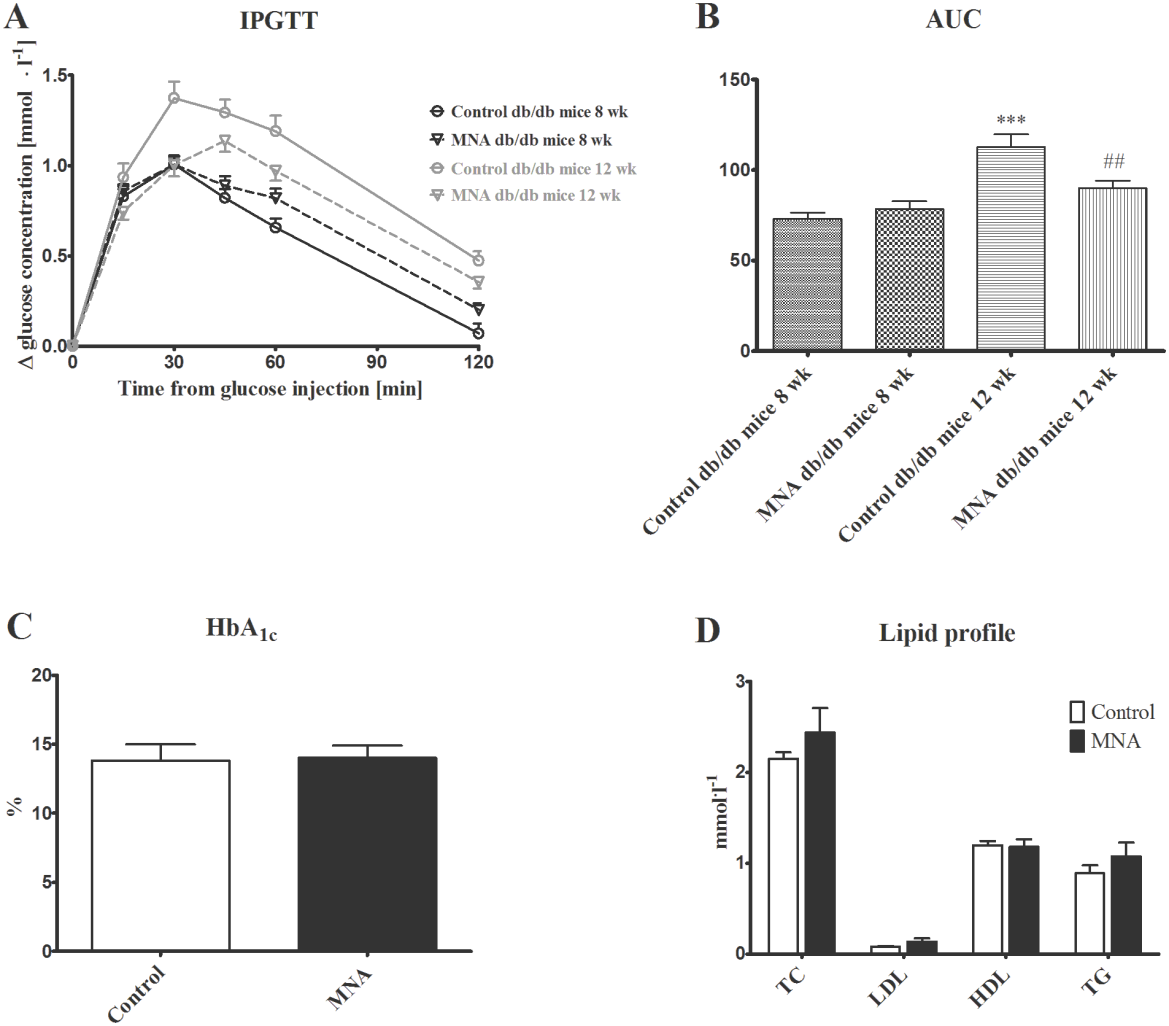
Effect of 4 weeks treatment with MNA on diabetic profile. Intraperitoneal glucose tolerance test (IPGTT) (A) (n = 18–20), blood glucose area under the curve (AUC) for IPGTT (B) (n = 18–20), blood HbA_1c_ concentration (C) (n = 7), lipid profile (D) (n = 7). TC (total cholesterol), LDL (low-density lipoprotein), HDL (high-density lipoprotein), and TG (triglycerides). MNA-treated db/db mice were supplemented with MNA in drinking water for 4 weeks at a dose of 100 mg·kg^-1^. The effects of MNA on blood HbA_1c_ concentration and lipid profile were evaluated in sedentary db/db mice. Data are presented as the mean ±SEM. Statistical analysis was performed using the Mann-Whitney test or unpaired t-test depending on the results of the normality test. ***P<0.001 vs. control db/db mice at 8 weeks of age, ##P<0.01 vs. control db/db mice at 12 weeks of age.

**Fig 3:**
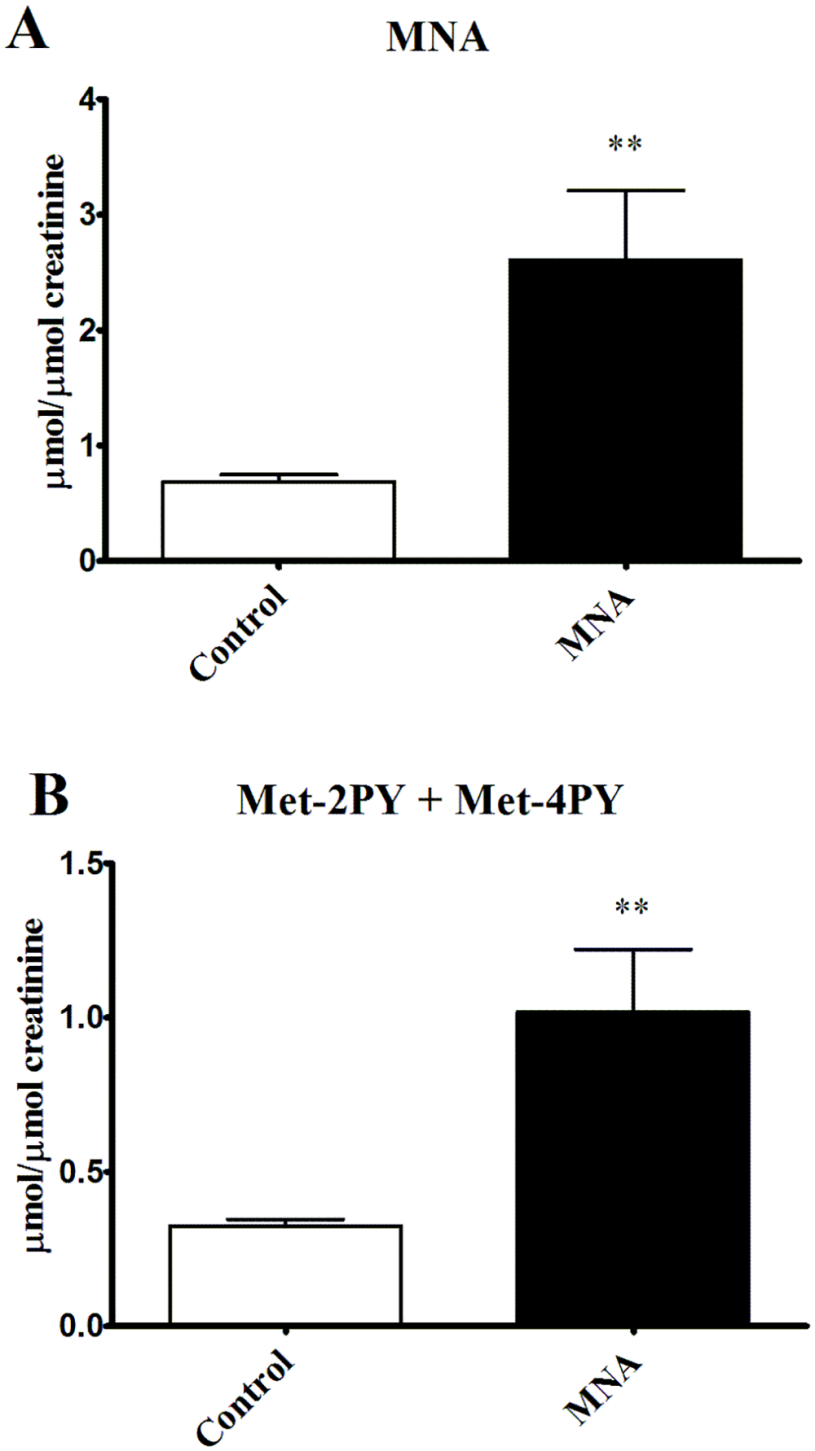
Concentrations of MNA and its metabolites in urine in untreated and MNA-treated db/db mice. To determine the effect of 4 weeks MNA supplementation on the urine concentrations of MNA and its metabolites, urine samples were collected from sedentary untreated and MNA-treated db/db mice for 24 h, one day before the endurance running test. Data are presented as the mean ±SEM. Statistical analysis was performed using the Mann-Whitney test or unpaired t-test depending on the results of the normality test. **P<0.01 (n = 7–6).

At the beginning of current experiment 8-week-old db/db mice were diabetic, as evidenced by higher blood glucose area under the curve (AUC) in IPGTT in comparison with wild-type mice (75.70±2.76 vs. 60.58±4.10 in wild-type mice, P<0.05, n = 39–6). As shown in Fig 2A and 2B, 4 weeks of treatment with MNA (100 mg·kg^-1^) significantly reduced insulin resistance in 12-week-old db/db mice as compared to 12-week-old untreated db/db mice (90.2±4.0 vs. 112.9±6.9, respectively, P<0.01, n = 18). However, there were no differences between MNA-treated and MNA-untreated 12-week-old db/db mice as regards to HbA_1c_ concentration (14.02±0.87 vs. 13.81±1.17%, respectively, n = 7, Fig 2C) and fasting glucose concentration (1.545±0.087 vs. 1.410±0.092 mmol. l^-1^, respectively, n = 18). Furthermore, MNA treatment did not affect the lipid profile (Fig 2D), blood cell count or the haematocrit (HCT) and haemoglobin (HGB) concentrations (Table 1). Finally, treatment with MNA did not diminish but rather tended to increase body weight gain in db/db mice (3.97±0.67 vs. 6.03±1.10 g for MNA-treated group, n = 10). In MNA-treated mice MNA and Met-2PY + Met-4PY concentrations in the urine were elevated by approximately four-fold and three-fold, respectively, as compared with untreated mice (e.g. the MNA concentration increased from 0.687±0.065 to 2.606±0.602 μmol. μmol creatinine^-1^, P<0.01, n = 7–6, Fig 3).

**Table 1:** Blood cell count in untreated and MNA-treated db/db mice.

	Sedentary		Exercised	
	Control	MNA	Control	MNA
**WBC** [K · μl^-1^]	1.367±0.226	2.350±0.343	3.150±0.452*	2.100±0.298
**RBC** [M · μl^-1^]	11.09±0.163	11.17±0.327	11.28±0.169	11.58±0.164
**HGB** [g · dL^-1^]	16.17±0.199	16.11±0.560	16.48±0.378	17.07±0.214
**HCT** [%]	56.83±1.014	56.83±1.978	60.11±1.265	61.98±0.954#
**PLT** [K · μl^-1^]	1315±68.78	1465±156.6	1252±46.85	1282±68.45

WBC, white blood cells; RBC, red blood cells; HGB, haemoglobin; HCT, haematocrit; PLT, platelets. Data are expressed as mean ±SEM. Statistical analysis was performed using the Mann-Whitney test or unpaired t-test depending on the results of the normality test.

*P<0.05 vs. sedentary control group

^#^P<0.05 vs. sedentary MNA group (n = 6–11)

### Effect of MNA treatment on exercise capacity in diabetic (db/db) mice

**Fig 4:**
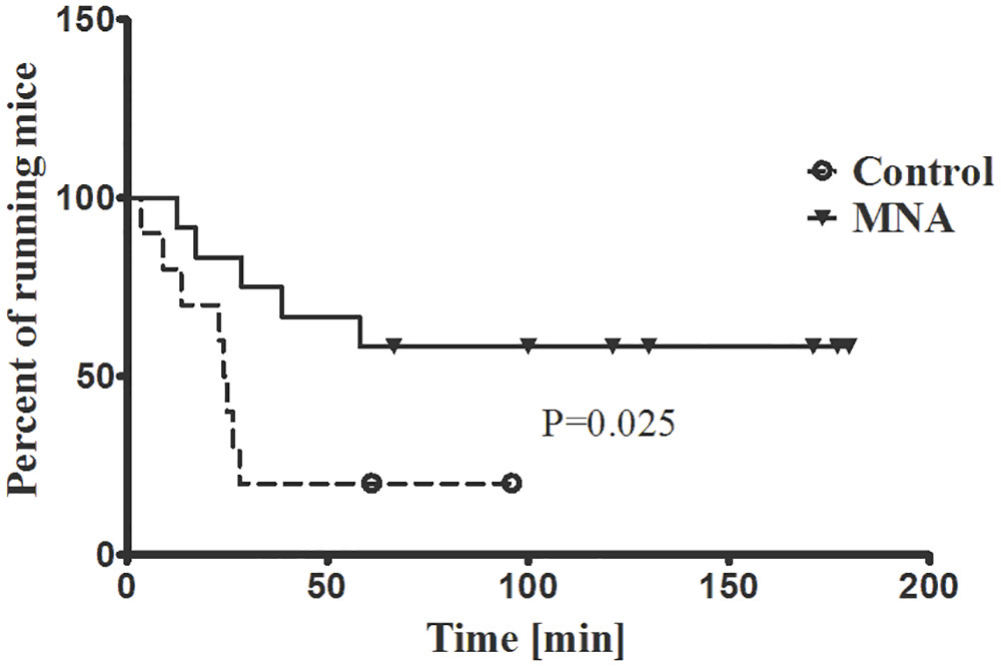
Effect of MNA treatment on exercise capacity in db/db mice. Untreated db/db mice and db/db mice treated with MNA for 4 weeks were subjected to endurance running at 8 m·min^-1^ on a treadmill with 5° inclination. The graph shows Kaplan-Meier curves of running mice in percentage for the two experimental groups: untreated mice (dashed line) and MNA-treated mice (solid line). Censored observations are marked with circles or triangles for the untreated and MNA-treated groups, respectively. Running time analysis was performed with Kaplan-Meier estimation using the log-rank Mantel-Cox test (n = 10–12).

**Fig 5:**
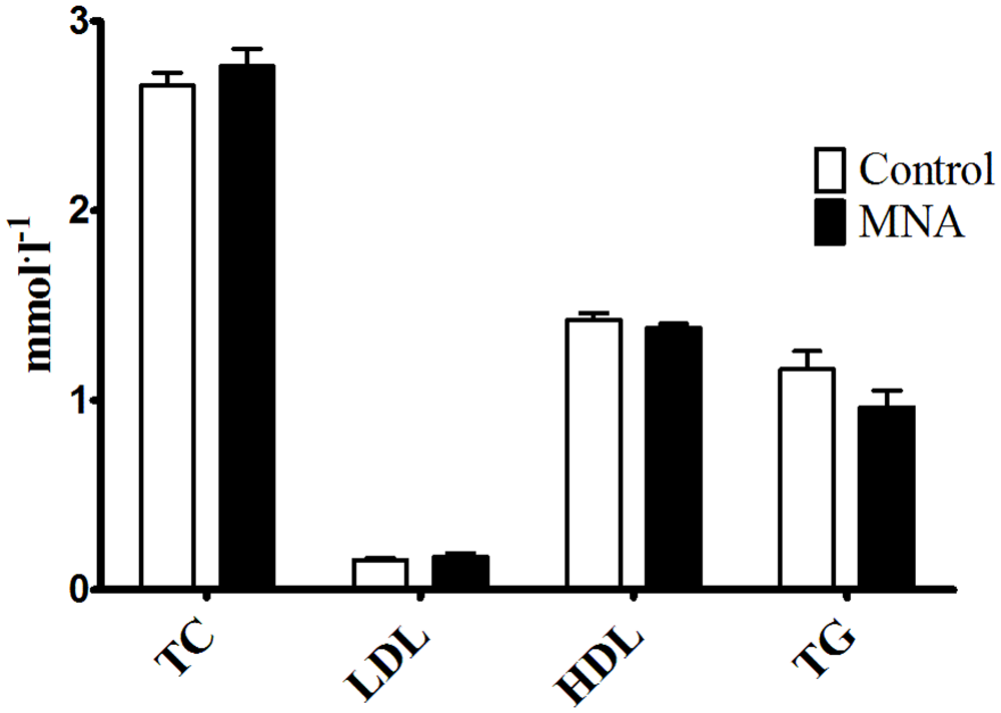
Post-exercise lipid profile in untreated and MNA-treated db/db mice. TC (total cholesterol), LDL (low-density lipoprotein), HDL (high-density lipoprotein), and TG (triglycerides). To determine the MNA effect on post-exercise lipid profile, blood samples were taken from exercised untreated and MNA-treated db/db mice. Data are presented as the mean ±SEM. Statistical analysis was performed using the Mann-Whitney test or unpaired t-test depending on the results of the normality test (n = 10–11).

**Fig 6:**
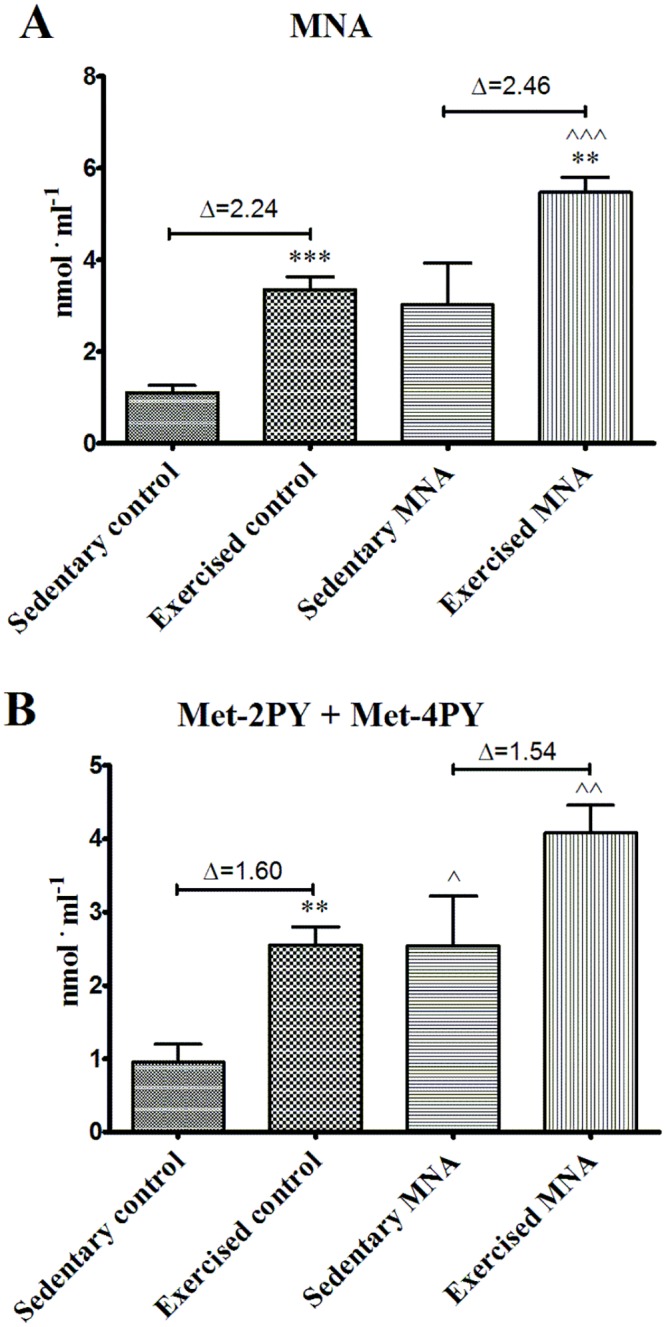
Post-exercise concentrations of MNA and its metabolites in plasma from untreated and MNA-treated db/db mice. Delta (Δ) denotes the difference between the mean concentration of a given metabolite determined at rest in the sedentary group and in the exercised group of mice after completing the fatiguing run. Data are presented as the mean ±SEM. Statistical analysis was performed using the Mann-Whitney test or unpaired t-test depending on the results of the normality test. **P<0.01, ***P<0.001 vs. corresponding sedentary group; ^P<0.05, ^^P<0.01, ^^^P<0.001 vs. corresponding control group (n = 7–11).

As shown in Fig 4, db/db mice treated with MNA displayed improved exercise capacity as evidenced by the prolonged endurance running time (P = 0.025). Improved exercise capacity by MNA was not related to changes in post-exercise blood cell count, HCT, HGB, total cholesterol (TC), low-density lipoprotein (LDL), high-density lipoprotein (HDL) or triglycerides (TG) concentrations (Table 1, Fig 5). However, HCT significantly increased in post-exercise MNA-treated db/db mice (from 56.83±1.978 to 61.98±0.954%, P<0.05, n = 6–9), while post-exercise leukocytosis was substantially inhibited (for untreated mice, 3.150±0.452 vs. 1.367±0.226 K. μl^-1^, P<0.05, n = 8–6, and for MNA-treated mice, 2.100±0.298 vs. 2.350±0.343 vs. K. μl^-1^, for post-exercise and sedentary groups, respectively, n = 10–6, Table 1). Endurance exercise resulted in a substantial increase in MNA plasma concentration in untreated db/db mice (from 1.115±0.156 to 3.351±0.280 nmol·ml^-1^, P<0.001, n = 7–10, Fig 6A). In MNA-treated db/db mice, the post-exercise increase in MNA plasma concentration was also significant (from 3.019±0.918 to 5.479±0.328 nmol·ml^-1^, P<0.01, n = 7–11). Interestingly, the relative exercise-induced increase in the plasma MNA concentration was similar in both the MNA-treated and untreated db/db mice (Δ = 2.46 and Δ = 2.24 nmol·ml^-1^ in MNA-treated and untreated groups, respectively), although the pre-exercise MNA plasma concentration was approximately 2.5-fold higher in MNA-treated db/db mice as compared to untreated db/db mice (3.019±0.918 vs. 1.115±0.156 nmol·ml^-1^, P = 0.063, n = 7, Fig 6A). The pattern of exercise-induced changes in the plasma concentration of the MNA metabolites (Met-2PY and Met-4PY) was similar to that of MNA (Fig 6B).

### Effect of MNA treatment on exercise-induced PGI_2_ release and nitrate utilization

**Fig 7:**
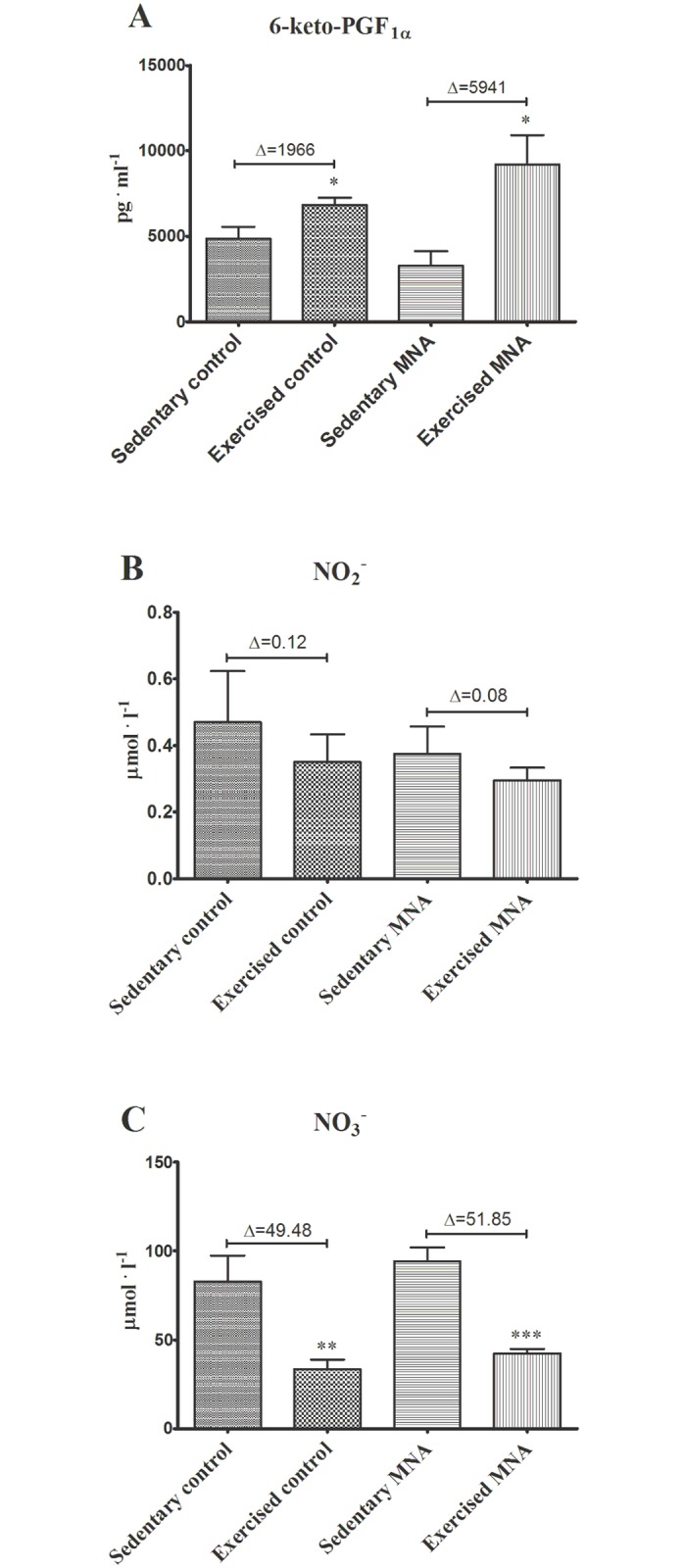
Post-exercise plasma concentration of 6-keto-PGF_1α_ (A) nitrite (B) and nitrate (C) in untreated and MNA-treated db/db mice. Delta (Δ) denotes the difference between the mean concentration of a given metabolite determined at rest in the sedentary group and in the exercised group of mice after completing the fatiguing run. Data are presented as the mean ±SEM. Statistical analysis was performed using the Mann-Whitney test or unpaired t-test depending on the results of the normality test. *P<0.05, **P<0.01, ***P<0.001 vs. corresponding sedentary group (n = 6–12).

Endurance exercise induced a significant increase in 6-keto-PGF_1α_ plasma concentration in both untreated (4862±684.9 vs. 6828±419 pg·ml^-1^, P<0.05, n = 7–10) and MNA-treated db/db mice (3263±860.7 vs. 9204±1716 vs. pg·ml^-1^, P<0.05, n = 7–12) (Fig 7A). The post-exercise 6-keto-PGF_1α_ plasma concentration in MNA-treated db/db mice was not significantly different from untreated db/db mice, however, the post-exercise increase in 6-keto-PGF_1α_ (Δ6-keto-PGF_1α_) plasma concentration was higher in MNA-treated animals (Δ6-keto-PGF_1α_ = 5941 in MNA group vs. 1966 pg·ml^-1^ in control group, Fig 7A). There were no significant differences in nitrite and nitrate plasma concentrations between sedentary untreated and sedentary MNA-treated db/db mice (Fig 7B and 7C), although in untreated and MNA-treated mice, the post-exercise plasma concentrations of nitrate were significantly lower (Fig 7C). The post-exercise fall in plasma nitrate concentration was similar for both untreated and MNA-treated groups (for untreated group ΔNO_3_^-^ = 49.48 and for MNA-treated group Δ = 51.85, Fig 7C). The post-exercise concentration of nitrite only tended to fall in the untreated and MNA-treated groups, and there was no difference between groups (Fig 7B).

## Discussion

In the present work, we demonstrated for the first time that long-term supplementation with MNA improved endurance exercise capacity in diabetic mice. We suggest that this MNA-induced effect could be linked to PGI_2_ and improvement of insulin sensitivity, but not to direct anti-diabetic effects of MNA.

It has been previously reported that anti-thrombotic, anti-inflammatory and gastroprotective effects of MNA are mediated by PGI_2_ [3–5]. In the present study, we demonstrated that there were no statistically significant differences in post-exercise 6-keto-PGF_1α_ plasma concentrations between untreated and MNA-treated mice (Fig 7A). However, increases in plasma 6-keto-PGF_1α_ concentrations induced by exercise were remarkably greater in the MNA-treated group (the magnitude of the Δ increase was augmented by approximately 45%). On the other hand, MNA did not appear to have any effects on fasting glucose, HbA_1c_ concentrations or lipid profile, although an increase in insulin sensitivity was observed (Fig 2A–2C). At the start of current experiment 8-week-old db/db mice were diabetic, as evidenced by significant insulin resistance in comparison with wild-type mice. The lack of the effect of MNA on HbA_1c_ concentrations but the apparent effect on insulin resistance may be due to the short period of MNA supplementation. It may have been long enough to improve insulin sensitivity but not HbA_1c_. On the other hand, changes in HbA_1c_ concentrations caused by anti-diabetic treatment, e.g. pioglitazone, has been shown to occur after 4-week-long treatment in a rat model of diabetes [19], suggesting that MNA may affect insulin resistance rather than directly causing hypoglycemia, in line with the previous work by Watala et al. [20]. Altogether, our results suggest that the MNA-induced effect on exercise capacity could perhaps be partially linked to the improvement in insulin sensitivity, although it was most likely associated with PGI_2_-mediated mechanisms.

It is commonly accepted that exercise alone leads to an increase in PGI_2_ release as assessed by measuring stable metabolites in human plasma [7, 21] and urine [8]. Moreover, the magnitude of the increase in PGI_2_ concentration in the interstitial muscle fluid in response to exercise depends on exercise intensity [22]. Recent data suggest that PGI_2_ plays a role in the regulation of exercise capacity, as PGI_2_ release in response to exercise was positively correlated with V’O_2max_ in healthy men [9]. Moreover, the training-induced increase in V’O_2max_ was accompanied by increased PGI_2_ release during exercise in the responders group. Interestingly, in the group of subjects in whom no increase was found in V’O_2max_ after training (non-responders), no changes were observed in the exercise-induced release of PGI_2_ after training [23]. These findings strongly suggest that PGI_2_ plays a role in the training-induced regulation of V’O_2max_ in humans. It is also well known that PGI_2_ alone or its stable analogue iloprost are able to increase exercise capacity in patients with pulmonary hypertension [24, 25] and stable angina pectoris [26]. It is important to add that, such individuals unaccustomed to habitual physical activities who undertake vigorous exercise have a 50-fold increase in the risk of sudden death and a 100-fold increase in the risk of acute myocardial infarction [11]. For example, patients suffering from diabetes have impaired ability to release PGI_2_ during exercise [10] and are characterized by high cardiovascular risk during vigorous exercise [11]. Accordingly, the magnitude of exercise-induced PGI_2_ release is an important factor that determines exercise tolerance, as well as the cardiovascular risk of vigorous exercise [9]. PGI_2_-mediated safeguarding effects of MNA on exercise capacity may rely on the protection of coronary, pulmonary and peripheral microcirculation through the inhibition of platelets from forming aggregates during vigorous exercise or/and the improvement of cardiac output [9].

In the present study, we did not find any differences in post-exercise 6-keto-PGF_1α_ plasma concentrations between untreated and MNA-treated mice (Fig 7A). However, the relative values of Δ increase in plasma 6-keto-PGF_1α_ concentration induced by exercise, were remarkably greater in the MNA-treated group. It might be that the post-exercise peak of the plasma 6-keto-PGF_1α_ concentration reflecting PGI_2_ production occurs immediately after the end of exercise, and quickly declines. This could explain why we did not see any evidence for the release of PGI_2_ by MNA after exercise in the blood taken within 3–5 minutes, the period of time needed for anaesthesia (pentobarbital) and blood sampling. Catheter placement and instant post-exercise sampling would be required to confirm the effect of MNA on PGI_2_ release during exercise. Additionally, in contrast to humans, measurement of pre-exercise (baseline) and post-exercise plasma concentrations of 6-keto-PGF_1α_ in the same mice is technically challenging. It is also important to note that plasma 6-keto-PGF_1α_ might be quickly metabolized during exercise into 2,3-dinor-6-keto-PGF_1α_, and excreted into the urine. It is not possible to collect urine from mice during and immediately after endurance running in order to compare the urine concentrations of 2,3-dinor-6-keto-PGF_1α_ between MNA-treated and untreated mice after endurance running.

In contrast to the increase in the post-exercise concentration of 6-keto-PGF_1α_, the concentration of nitrate decreased. Interestingly, this exercise-induced response was not modified by MNA treatment. Our data seem to be discordant with data from other studies showing an increase or preservation of post-exercise plasma nitrite and nitrate concentrations in healthy humans [14–16].

It is well established that under hypoxic conditions, nitrite and nitrate can be reduced back to NO *in vivo*, thereby being an alternative source of NO for the NOS-dependent pathway [12]. These conditions, with lower oxygen tension, occur in skeletal muscle during exhaustive exercise [13]. In particular, NO_2_^-^-derived NO may be important in the setting of impaired endothelial NO production, as was the case for db/db mice at the age of 12 weeks [27], that were used in the present experiments.

Our data showing a pronounced fall in the post-exercise plasma concentration of nitrate may suggest that exercise in db/db mice with endothelial dysfunction may indeed activate the reductive pathway of NO generation, i.e. NO_3_^-^-NO_2_^-^-NO. Accordingly, it appears as though exercise-induced NO formation in diabetic mice was mainly sustained by this reductive pathway, not by endothelial NO production, which was obviously impaired in diabetic mice. If so, it seems obvious that MNA did not modify the post-exercise fall in the plasma concentration of nitrate.

It is well known that the total number of white blood cells is increasing after exercise [28–30]. This phenomenon most likely occurs in response to exercise-induced skeletal muscle damage. The post-exercise increase in neutrophil count is correlated with increases in markers of skeletal muscle damage, such as plasma myoglobin concentration and plasma creatine kinase activity [30]. This notion is also supported by reports showing, leukocyte accumulation in exercised muscles, which was associated with a local inflammatory response resulting from exercise-induced muscle damage [31]. Interestingly, the function of the immune system is suppressed by acute bouts of endurance exercise, increasing the susceptibility to upper respiratory illness [32]. On the other hand, leukocytosis may be caused by sympathetic system-mediated mechanisms [33]. In the present study, we demonstrated that MNA decreased post-exercise leukocytosis, suggesting anti-inflammatory or/and anti-sympathetic profile of MNA activity.

Schmeisser et al. [34] has suggested that MNA increased the speed of crawling in nematodes *C*. *elegans* by reactive oxygen species (ROS)-dependent mechanism. This group also discovered that MNA, generated through the sirtuin-dependent pathway, extended the lifespan of nematodes by the induction of ROS and subsequent hydrogen peroxide generation by an aldehyde oxidase, GAD-3 [34]. It still remains to be established whether ROS-dependent mechanisms are involved in the MNA-induced effects on exercise capacity in db/db mice.

In conclusion, in the present work, we demonstrated for the first time that long-term supplementation with MNA results in an improvement of exercise capacity in diabetic mice, most likely by PGI_2_-dependent pathways. However, the underlying mechanisms need to be further investigated. As the release of PGI_2_ in response to exercise appears to play a role in the regulation of exercise capacity [9], the impairment of exercise-induced PGI_2_ release may lead to an increase in cardiovascular risk during high-intensity exercise. We assume that MNA-dependent stimulation of PGI_2_ release not only improves exercise capacity in pathological states with impaired endothelial function and compromised exercise tolerance but also protects the coronary, pulmonary and peripheral microcirculation against the formation of platelet microaggregates, thereby preserving adequate tissue perfusion in skeletal muscle, as well as sustaining optimal cardiac output. Safeguarding the pro-aggregatory platelet response seems to be crucial for the safety of exercise in patients at high cardiovascular risk. In summary, we suggest that MNA affords protection against cardiovascular risk caused by long moderate-intensity exercise sessions as implemented in the current study, but may also protect diabetic or cardiovascular patients with impaired endothelial function during exercise of higher intensity and shorter duration. Further studies in humans are warranted to translate our findings to humans.
